# (*E*)-1-(4-Fluoro-2-hy­droxy­phen­yl)-3-(2,3,4-tri­meth­oxy­phen­yl)prop-2-en-1-one

**DOI:** 10.1107/S2414314620000711

**Published:** 2020-01-24

**Authors:** Miri Yoo, Dongsoo Koh

**Affiliations:** a Department of Applied Chemistry, Dongduk Women’s University, Seoul 136-714, Republic of Korea; University of Toronto, Canada

**Keywords:** crystal structure, chalcone, C—H⋯O hydrogen bonds, dihedral angle

## Abstract

In the title mol­ecule, C_18_H_17_FO_5_, the conformation about the C=C bond of the central enone group is *trans*. The dihedral angle between the benzene rings is 13.08 (3)°. The hy­droxy group attached to the benzene ring is involved in an intra­molecular O—H⋯O hydrogen bond. In the crystal, weak C—H⋯O hydrogen bonds link the mol­ecules into chains along [001].

## Abstract 2

The crystal structure of a biologically important pharmacophore containing chalcone is reported.

## Graphical abstract

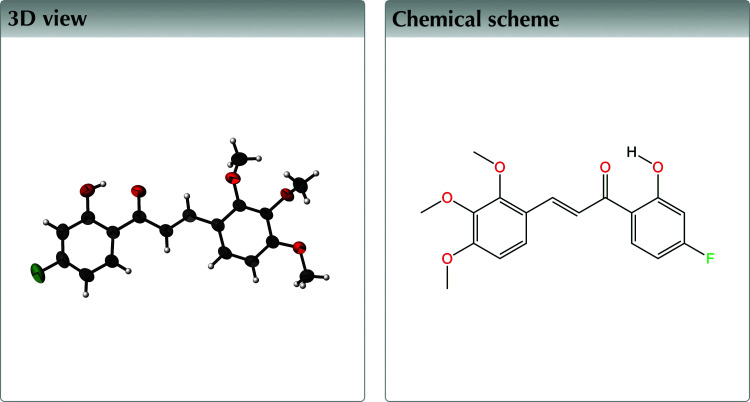

## Structure description

Chalcones are *α*,*β*-unsaturated carbonyl (enone) compounds, which connect two aromatic rings. Especially when they have a hydroxyl group at the *ortho* position of an aromatic ring adjacent to the carbonyl group, they play important roles as precursors to form other flavonoids such as flavones, flavanones, flavonols and isoflavones (Marais *et al.*, 2005 ▸). A variety of chlacones have been isolated from natural sources and synthesized because they have shown wide spectrum of biological activities against various diseases according to a recent review (Zhuang *et al.*, 2017 ▸). In a continuation of our research inter­ests to prepare new chalcones that show broad range of biological activities (Gil *et al.*, 2018 ▸, Park *et al.*, 2018 ▸), the crystal structure of title compound has been determined.

**Figure 1:**
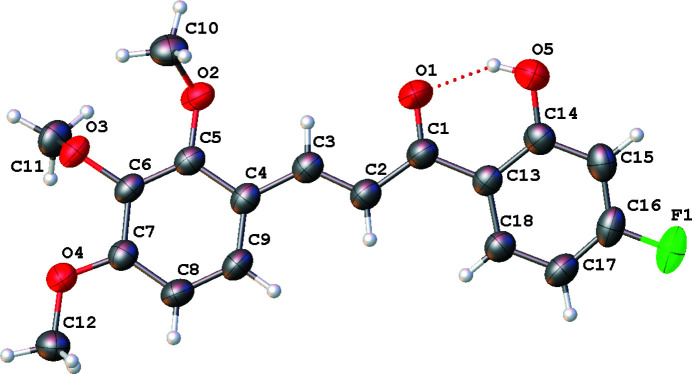
The mol­ecular structure of the title compound, showing the atom-labelling scheme with displacement ellipsoids drawn at the 30% probability level. The intra­molecular hydrogen bond is shown as a dashed line.

**Figure 2:**
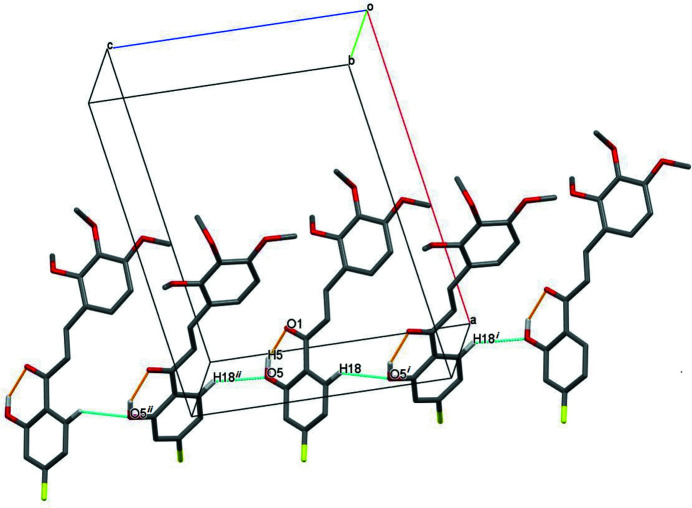
Part of the crystal structure with the intra­molecular O—H⋯O hydrogen bond and weak C—H⋯O hydrogen bonds shown as dashed lines. For the sake of clarity, only the H atoms involved in hydrogen bonds are shown [symmetry codes: (i) *x*, −*y* + 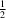 , *z* − 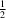 ; (ii) *x*, −*y* + 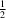 , *z* + 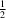 ].

The mol­ecular structure of the title compound is shown in Fig. 1 ▸. In the central enone group, the *trans* configuration of the C2=C3 double bond is confirmed by the C1—C2=C3—C4 torsion angle of −176.6 (2)°. An intra­molecular O5—H5⋯O1 hydrogen bond (Table 1 ▸) appears to cause the C1=O1 double bond [1.239 (2) Å] to be slightly longer than the normal value (Allen *et al.*, 1987 ▸). The dihedral angle between the two benzene rings is 13.08 (3)°. Among the meth­oxy groups attached to the C4 benzene ring, the meth­oxy group at the *meta* position is almost perpendicular to the benzene ring [C5—C6—O3—C11 = 86.3 (2)°] and the *para* meth­oxy group is almost coplanar with the ring [C6—C7—O4—C12 = 177.1 (2)°]. The meth­oxy group at the *ortho* position is rotated significantly from the ring plane [C4—C5—O2—C10 = −123.9 (2)°]. In the crystal, weak C—H⋯O hydrogen bonds link the mol­ecules into chains propagating along [001] (Table 1 ▸, Fig. 2 ▸).

## Synthesis and crystallization

To a solution of 1-(4-fluoro-2-hy­droxy­phen­yl)ethanone (309 mg, 2 mmol) in 40 ml of anhydrous ethanol was added 2,3,4-tri­meth­oxy­benzaldehyde (392 mg, 2 mmol) and the temperature was adjusted to around 275–277 K in an ice bath. To the cooled reaction mixture was added 3 ml of 40% aqueous KOH solution and the reaction mixture was stirred at room temperature for 20 h. After completion of the reaction (monitored by thin-layer chromatography), this mixture was poured into ice water (100 ml) and the resulting solution acidified with 6 *N* HCl solution until pH = 3 to produce a solid product. This solid was recrystallized from an ethanol solution to obtain single crystals of the title compound in 54% yield.

## Refinement

Crystal data, data collection and structure refinement details are summarized in Table 2 ▸.

## Supplementary Material

Crystal structure: contains datablock(s) I. DOI: 10.1107/S2414314620000711/lh4051sup1.cif

Structure factors: contains datablock(s) I. DOI: 10.1107/S2414314620000711/lh4051Isup2.hkl

Supporting information file. DOI: 10.1107/S2414314620000711/lh4051Isup3.cml

CCDC reference: 1979111

Additional supporting information:  crystallographic information; 3D view; checkCIF report

## Figures and Tables

**Table 1:** Hydrogen-bond geometry (Å, °)

*D*—H⋯*A*	*D*—H	H⋯*A*	*D*⋯*A*	*D*—H⋯*A*
O5—H5⋯O1	0.83	1.77	2.499 (2)	146
C18—H18⋯O5^i^	0.94	2.52	3.279 (2)	138

Symmetry code: (i) 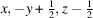 .

**Table 2:** Experimental details

Crystal data	
Chemical formula	C_18_H_17_FO_5_
*M*_r_	332.32
Crystal system, space group	Monoclinic, *P*2_1_/*c*
Temperature (K)	223
*a*, *b*, *c* (Å)	15.5801 (12), 8.3414 (6), 12.1298 (8)
β (°)	97.086 (3)
*V* (Å^3^)	1564.35 (19)
*Z*	4
Radiation type	Mo *K*α
μ (mm^−1^)	0.11
Crystal size (mm)	0.21 × 0.15 × 0.10

Data collection	
Diffractometer	PHOTON 100 CMOS
Absorption correction	Multi-scan (*SADABS*; Bruker, 2012 ▸)
*T*_min_, *T*_max_	0.706, 0.746
No. of measured, independent and observed [*I* > 2σ(*I*)] reflections	64701, 3904, 2539
*R*_int_	0.079
(sin θ/λ)_max_ (Å^−1^)	0.668

Refinement	
*R*[*F* ^2^ > 2σ(*F* ^2^)], *wR*(*F* ^2^), *S*	0.048, 0.125, 1.01
No. of reflections	3904
No. of parameters	221
H-atom treatment	H-atom parameters constrained
Δρ_max_, Δρ_min_ (e Å^−3^)	0.21, −0.21

Computer programs: *APEX2* and *SAINT* (Bruker, 2012 ▸), *SHELXTL* (Sheldrick, 2008 ▸), *SHELXL2014* (Sheldrick, 2015 ▸), *OLEX2* (Dolomanov *et al.*, 2009 ▸) and *publCIF* (Westrip, 2010 ▸).

## full crystallographic data

### Crystal data

**Table:**

C_18_H_17_FO_5_	*F*(000) = 696
*M**_r_* = 332.32	*D*_x_ = 1.411 Mg m^−^^3^
Monoclinic, *P*2_1_/*c*	Mo *K*α radiation, λ = 0.71073 Å
*a* = 15.5801 (12) Å	Cell parameters from 9964 reflections
*b* = 8.3414 (6) Å	θ = 2.8–25.8°
*c* = 12.1298 (8) Å	µ = 0.11 mm^−^^1^
β = 97.086 (3)°	*T* = 223 K
*V* = 1564.35 (19) Å^3^	Block, yellow
*Z* = 4	0.21 × 0.15 × 0.10 mm

### Data collection

**Table:**

PHOTON 100 CMOS diffractometer	2539 reflections with *I* > 2σ(*I*)
φ and ω scans	*R*_int_ = 0.079
Absorption correction: multi-scan (SADABS; Bruker, 2012)	θ_max_ = 28.4°, θ_min_ = 2.6°
*T*_min_ = 0.706, *T*_max_ = 0.746	*h* = −20→20
64701 measured reflections	*k* = −11→11
3904 independent reflections	*l* = −16→16

### Refinement

**Table:**

Refinement on *F*^2^	0 restraints
Least-squares matrix: full	Hydrogen site location: inferred from neighbouring sites
*R*[*F*^2^ > 2σ(*F*^2^)] = 0.048	H-atom parameters constrained
*wR*(*F*^2^) = 0.125	*w* = 1/[σ^2^(*F*_o_^2^) + (0.0473*P*)^2^ + 0.6183*P*] where *P* = (*F*_o_^2^ + 2*F*_c_^2^)/3
*S* = 1.01	(Δ/σ)_max_ = 0.001
3904 reflections	Δρ_max_ = 0.21 e Å^−^^3^
221 parameters	Δρ_min_ = −0.21 e Å^−^^3^

### Special details

**Table:**

Geometry. All esds (except the esd in the dihedral angle between two l.s. planes) are estimated using the full covariance matrix. The cell esds are taken into account individually in the estimation of esds in distances, angles and torsion angles; correlations between esds in cell parameters are only used when they are defined by crystal symmetry. An approximate (isotropic) treatment of cell esds is used for estimating esds involving l.s. planes.

### Fractional atomic coordinates and isotropic or equivalent isotropic displacement parameters (Å^2^)

**Table:**

	*x*	*y*	*z*	*U*_iso_*/*U*_eq_	
O1	0.92429 (10)	0.0434 (2)	0.67173 (13)	0.0787 (5)	
C1	0.95147 (12)	0.1327 (2)	0.60215 (15)	0.0465 (4)	
C2	0.89787 (11)	0.1589 (2)	0.49622 (15)	0.0448 (4)	
H2	0.9178	0.2247	0.4418	0.054*	
C3	0.82032 (11)	0.0890 (2)	0.47693 (15)	0.0433 (4)	
H3	0.8031	0.0297	0.5363	0.052*	
C4	0.75915 (10)	0.0924 (2)	0.37646 (14)	0.0406 (4)	
C5	0.67961 (11)	0.01064 (19)	0.37400 (14)	0.0387 (4)	
C6	0.62259 (10)	0.00211 (19)	0.27734 (14)	0.0384 (4)	
C7	0.64306 (11)	0.0776 (2)	0.18080 (15)	0.0415 (4)	
C8	0.71985 (12)	0.1619 (2)	0.18280 (16)	0.0487 (4)	
H8	0.7334	0.2144	0.1186	0.058*	
C9	0.77632 (11)	0.1685 (2)	0.27966 (15)	0.0473 (4)	
H9	0.8281	0.2263	0.2802	0.057*	
O2	0.65803 (8)	−0.04848 (15)	0.47252 (10)	0.0482 (3)	
C10	0.63936 (14)	−0.2150 (2)	0.47998 (17)	0.0563 (5)	
H10A	0.5893	−0.2413	0.4275	0.084*	
H10B	0.6274	−0.2400	0.5547	0.084*	
H10C	0.6887	−0.2770	0.4629	0.084*	
O3	0.54649 (7)	−0.08295 (14)	0.27272 (10)	0.0439 (3)	
C11	0.47618 (11)	0.0049 (2)	0.30660 (18)	0.0553 (5)	
H11A	0.4884	0.0307	0.3849	0.083*	
H11B	0.4239	−0.0591	0.2942	0.083*	
H11C	0.4682	0.1032	0.2638	0.083*	
O4	0.58278 (8)	0.06284 (15)	0.09020 (10)	0.0483 (3)	
C12	0.59864 (13)	0.1441 (2)	−0.00904 (16)	0.0544 (5)	
H12A	0.6038	0.2583	0.0054	0.082*	
H12B	0.5510	0.1248	−0.0669	0.082*	
H12C	0.6519	0.1045	−0.0330	0.082*	
C13	1.03648 (11)	0.2090 (2)	0.62893 (14)	0.0396 (4)	
C14	1.08778 (12)	0.1692 (2)	0.72906 (14)	0.0458 (4)	
C15	1.16780 (12)	0.2411 (2)	0.75754 (16)	0.0520 (5)	
H15	1.2027	0.2136	0.8237	0.062*	
C16	1.19430 (12)	0.3521 (2)	0.68738 (17)	0.0524 (5)	
C17	1.14761 (12)	0.3949 (2)	0.58816 (17)	0.0511 (5)	
H17	1.1687	0.4717	0.5415	0.061*	
C18	1.06905 (11)	0.3214 (2)	0.55947 (15)	0.0444 (4)	
H18	1.0364	0.3474	0.4914	0.053*	
O5	1.06213 (10)	0.06119 (19)	0.80053 (11)	0.0663 (4)	
H5	1.0118	0.0325	0.7789	0.099*	
F1	1.27125 (7)	0.42519 (16)	0.71683 (12)	0.0763 (4)	

### Atomic displacement parameters (Å^2^)

**Table:**

	*U*^11^	*U*^22^	*U*^33^	*U*^12^	*U*^13^	*U*^23^
O1	0.0693 (10)	0.1025 (13)	0.0620 (9)	−0.0386 (9)	−0.0006 (7)	0.0286 (9)
C1	0.0466 (10)	0.0478 (10)	0.0461 (10)	−0.0059 (8)	0.0095 (8)	−0.0009 (8)
C2	0.0420 (10)	0.0458 (10)	0.0476 (10)	−0.0037 (8)	0.0097 (8)	−0.0011 (8)
C3	0.0438 (10)	0.0410 (9)	0.0466 (10)	−0.0025 (8)	0.0109 (8)	−0.0047 (8)
C4	0.0382 (9)	0.0350 (9)	0.0497 (10)	−0.0018 (7)	0.0098 (7)	−0.0059 (7)
C5	0.0398 (9)	0.0323 (8)	0.0457 (9)	0.0000 (7)	0.0116 (7)	−0.0026 (7)
C6	0.0351 (8)	0.0303 (8)	0.0513 (10)	−0.0026 (7)	0.0113 (7)	−0.0024 (7)
C7	0.0404 (9)	0.0342 (9)	0.0500 (10)	−0.0004 (7)	0.0058 (8)	−0.0004 (7)
C8	0.0493 (11)	0.0470 (10)	0.0511 (11)	−0.0080 (8)	0.0114 (8)	0.0073 (8)
C9	0.0407 (10)	0.0451 (10)	0.0573 (11)	−0.0096 (8)	0.0101 (8)	−0.0002 (8)
O2	0.0555 (8)	0.0440 (7)	0.0476 (7)	−0.0117 (6)	0.0156 (6)	−0.0007 (6)
C10	0.0649 (13)	0.0420 (10)	0.0631 (12)	0.0024 (9)	0.0129 (10)	0.0091 (9)
O3	0.0372 (6)	0.0367 (6)	0.0587 (8)	−0.0067 (5)	0.0099 (5)	−0.0018 (5)
C11	0.0367 (10)	0.0583 (12)	0.0718 (13)	0.0030 (9)	0.0097 (9)	0.0003 (10)
O4	0.0483 (7)	0.0467 (7)	0.0491 (7)	−0.0092 (6)	0.0029 (6)	0.0060 (6)
C12	0.0595 (12)	0.0532 (12)	0.0501 (11)	−0.0050 (9)	0.0055 (9)	0.0075 (9)
C13	0.0403 (9)	0.0390 (9)	0.0408 (9)	−0.0008 (7)	0.0104 (7)	−0.0068 (7)
C14	0.0506 (11)	0.0470 (10)	0.0405 (9)	−0.0006 (8)	0.0083 (8)	−0.0064 (8)
C15	0.0484 (11)	0.0561 (12)	0.0495 (10)	0.0023 (9)	−0.0015 (9)	−0.0126 (9)
C16	0.0375 (10)	0.0525 (11)	0.0670 (13)	−0.0040 (8)	0.0058 (9)	−0.0188 (10)
C17	0.0444 (10)	0.0472 (10)	0.0637 (12)	−0.0054 (8)	0.0147 (9)	−0.0005 (9)
C18	0.0407 (9)	0.0450 (10)	0.0482 (10)	−0.0003 (8)	0.0088 (8)	−0.0002 (8)
O5	0.0720 (10)	0.0787 (10)	0.0467 (8)	−0.0169 (8)	0.0016 (7)	0.0116 (7)
F1	0.0467 (7)	0.0820 (9)	0.0978 (10)	−0.0187 (6)	−0.0004 (6)	−0.0190 (8)

### Geometric parameters (Å, º)

**Table:**

O1—C1	1.239 (2)	C10—H10C	0.9700
C1—O1	1.239 (2)	O3—C11	1.420 (2)
C1—C2	1.460 (3)	C11—H11A	0.9700
C1—C13	1.469 (2)	C11—H11B	0.9700
C2—C3	1.336 (2)	C11—H11C	0.9700
C2—H2	0.9400	O4—C12	1.429 (2)
C3—C4	1.452 (2)	C12—H12A	0.9700
C3—H3	0.9400	C12—H12B	0.9700
C4—C9	1.389 (2)	C12—H12C	0.9700
C4—C5	1.412 (2)	C13—C18	1.397 (2)
C5—O2	1.372 (2)	C13—C14	1.409 (2)
C5—C6	1.383 (2)	C14—O5	1.345 (2)
C6—O3	1.3768 (19)	C14—C15	1.388 (3)
C6—C7	1.400 (2)	C15—C16	1.356 (3)
C7—O4	1.360 (2)	C15—H15	0.9400
C7—C8	1.386 (2)	C16—F1	1.353 (2)
C8—C9	1.379 (3)	C16—C17	1.374 (3)
C8—H8	0.9400	C17—C18	1.375 (2)
C9—H9	0.9400	C17—H17	0.9400
O2—C10	1.424 (2)	C18—H18	0.9400
C10—H10A	0.9700	O5—H5	0.8300
C10—H10B	0.9700		
			
O1—C1—C2	118.90 (17)	H10B—C10—H10C	109.5
O1—C1—C2	118.90 (17)	C6—O3—C11	114.41 (13)
O1—C1—C13	118.98 (17)	O3—C11—H11A	109.5
O1—C1—C13	118.98 (17)	O3—C11—H11B	109.5
C2—C1—C13	122.12 (16)	H11A—C11—H11B	109.5
C3—C2—C1	119.70 (17)	O3—C11—H11C	109.5
C3—C2—H2	120.2	H11A—C11—H11C	109.5
C1—C2—H2	120.2	H11B—C11—H11C	109.5
C2—C3—C4	128.64 (17)	C7—O4—C12	117.62 (13)
C2—C3—H3	115.7	O4—C12—H12A	109.5
C4—C3—H3	115.7	O4—C12—H12B	109.5
C9—C4—C5	117.57 (16)	H12A—C12—H12B	109.5
C9—C4—C3	122.92 (15)	O4—C12—H12C	109.5
C5—C4—C3	119.47 (16)	H12A—C12—H12C	109.5
O2—C5—C6	121.26 (15)	H12B—C12—H12C	109.5
O2—C5—C4	117.64 (15)	C18—C13—C14	117.93 (16)
C6—C5—C4	120.89 (15)	C18—C13—C1	122.82 (16)
O3—C6—C5	121.40 (15)	C14—C13—C1	119.25 (16)
O3—C6—C7	118.78 (15)	O5—C14—C15	117.28 (17)
C5—C6—C7	119.80 (15)	O5—C14—C13	122.11 (16)
O4—C7—C8	124.65 (16)	C15—C14—C13	120.61 (17)
O4—C7—C6	115.41 (14)	C16—C15—C14	118.20 (18)
C8—C7—C6	119.93 (16)	C16—C15—H15	120.9
C9—C8—C7	119.58 (17)	C14—C15—H15	120.9
C9—C8—H8	120.2	F1—C16—C15	118.06 (18)
C7—C8—H8	120.2	F1—C16—C17	118.05 (19)
C8—C9—C4	122.19 (16)	C15—C16—C17	123.89 (17)
C8—C9—H9	118.9	C16—C17—C18	117.68 (18)
C4—C9—H9	118.9	C16—C17—H17	121.2
C5—O2—C10	118.67 (14)	C18—C17—H17	121.2
O2—C10—H10A	109.5	C17—C18—C13	121.66 (18)
O2—C10—H10B	109.5	C17—C18—H18	119.2
H10A—C10—H10B	109.5	C13—C18—H18	119.2
O2—C10—H10C	109.5	C14—O5—H5	109.5
H10A—C10—H10C	109.5		
			
O1—O1—C1—C2	0.0 (2)	C6—C5—O2—C10	61.3 (2)
O1—O1—C1—C13	0.0 (3)	C4—C5—O2—C10	−123.86 (17)
O1—C1—C2—C3	1.2 (3)	C5—C6—O3—C11	86.36 (19)
O1—C1—C2—C3	1.2 (3)	C7—C6—O3—C11	−95.34 (19)
C13—C1—C2—C3	−178.72 (16)	C8—C7—O4—C12	−1.9 (2)
C1—C2—C3—C4	−176.61 (17)	C6—C7—O4—C12	177.06 (15)
C2—C3—C4—C9	3.0 (3)	O1—C1—C13—C18	−174.58 (19)
C2—C3—C4—C5	−179.42 (17)	O1—C1—C13—C18	−174.58 (19)
C9—C4—C5—O2	−172.57 (15)	C2—C1—C13—C18	5.3 (3)
C3—C4—C5—O2	9.7 (2)	O1—C1—C13—C14	4.9 (3)
C9—C4—C5—C6	2.3 (2)	O1—C1—C13—C14	4.9 (3)
C3—C4—C5—C6	−175.44 (15)	C2—C1—C13—C14	−175.23 (16)
O2—C5—C6—O3	−8.1 (2)	C18—C13—C14—O5	−179.35 (16)
C4—C5—C6—O3	177.25 (14)	C1—C13—C14—O5	1.1 (3)
O2—C5—C6—C7	173.64 (15)	C18—C13—C14—C15	0.5 (3)
C4—C5—C6—C7	−1.0 (2)	C1—C13—C14—C15	−178.97 (16)
O3—C6—C7—O4	1.9 (2)	O5—C14—C15—C16	−179.04 (17)
C5—C6—C7—O4	−179.78 (14)	C13—C14—C15—C16	1.1 (3)
O3—C6—C7—C8	−179.05 (15)	C14—C15—C16—F1	178.23 (16)
C5—C6—C7—C8	−0.7 (2)	C14—C15—C16—C17	−1.7 (3)
O4—C7—C8—C9	−179.89 (16)	F1—C16—C17—C18	−179.28 (16)
C6—C7—C8—C9	1.1 (3)	C15—C16—C17—C18	0.7 (3)
C7—C8—C9—C4	0.2 (3)	C16—C17—C18—C13	1.0 (3)
C5—C4—C9—C8	−1.9 (3)	C14—C13—C18—C17	−1.6 (3)
C3—C4—C9—C8	175.76 (17)	C1—C13—C18—C17	177.87 (17)

### Hydrogen-bond geometry (Å, º)

**Table:**

*D*—H···*A*	*D*—H	H···*A*	*D*···*A*	*D*—H···*A*
O5—H5···O1	0.83	1.77	2.499 (2)	146
C18—H18···O5^i^	0.94	2.52	3.279 (2)	138

Symmetry code: (i) *x*, −*y*+1/2, *z*−1/2.
